# [Corrigendum] The impact of metformin and salinomycin on transforming growth factor β-induced epithelial-to-mesenchymal transition in non-small cell lung cancer cell lines

**DOI:** 10.3892/ol.2025.15157

**Published:** 2025-06-25

**Authors:** Stefan Koeck, Arno Amann, Julia M. Huber, Gabriele Gamerith, Wolfgang Hilbe, Heinz Zwierzina

Oncol Lett 11: 2946–2952, 2016; DOI: 10.3892/ol.2016.4323

Following the publication of the above article, an interested reader drew to the authors’ attention that, for the scratch-wound assay experiments shown in Fig. 3 on p. 2948, the bottom two panels for the ‘Salinomycin, 1 μM’ experiments (the ‘-TGFβ’ and ‘+TGFβ’ panels) appeared to show the same cells, but with a different layer of edge tracking. Upon asking the authors for an explanation of this phenomenon, they were able to confirm that the underlying images in the bottom two panels were identical, and that the mistake occurred during the figure adaptation process for publication, given that an earlier version (prepared for an initial submission) did not contain this error. Furthermore, upon asking the authors how the dark areas in the abovementioned data panels differed, whereas the patterning of the cells appeared to be identical, they explained that the dark areas represented the regions initially covered by cells at the starting time point (0 h); the second images, captured 48 h later, were displayed as a grayscale background. The dark areas from the first image were then overlaid on to the second images to show the initial area covered with cells, and the same cellular (grayscale) image had been erroneously selected for the ‘Salinomycin, 1 μM/-TGFβ’ and ‘Salinomycin, 1 μM/+TGFβ’ experiments. [Note that ImageJ (v1.44; National Institutes of Health) was used to create the dark areas and to overlay the images.] The authors were also able to provide us with the raw data underlying the composite images shown in the various figure panels.

A revised version of Fig. 3, now showing the corrected data and all the experiments for the treatments of the A549 and HCC400 cell lines with different combinations/concentrations of metformin and salinomycin, is shown on the next page. The authors regret the error that occurred in the originally published version of this figure, although this did not grossly affect the results or the conclusions reported in this article. All the authors agree with the publication of this Corrigendum, and thank the Editor of *Oncology Letters* for granting them the opportunity to publish this; furthermore, they apologize to the readership for any inconvenience caused.

## Figures and Tables

**Figure 3.**
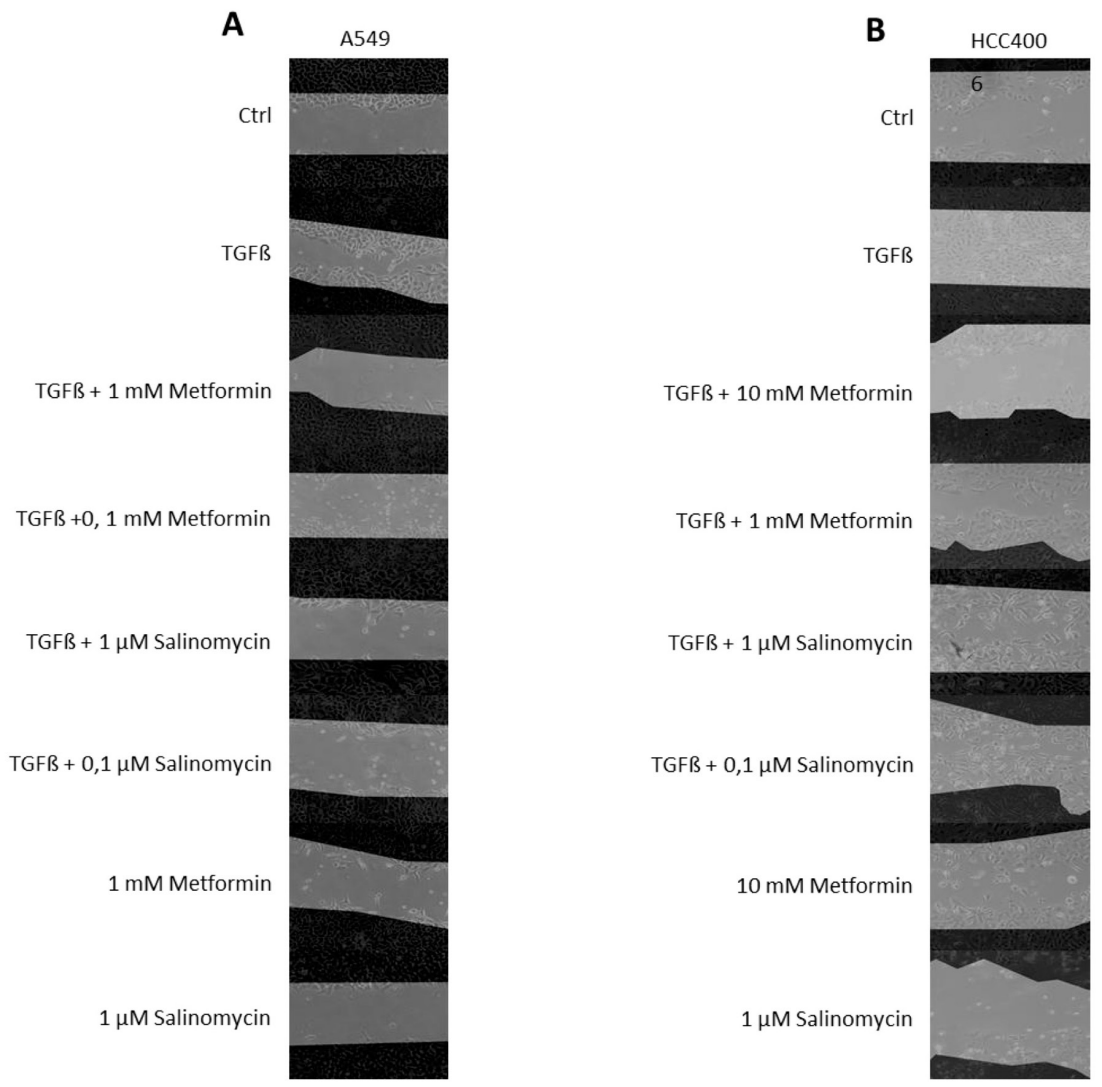
Representative images of scratch-wound assays conducted on the (A) A549 and (B) HCC4006 cell lines after 48 h. Images were captured at 0 h and 48 h after scratching. The dark areas represents the cell monolayer at the beginning of the experiment. TGFβ, transforming growth factor β.

